# The Relationship between Stress and Preclinical Dental Students' Performance: A Longitudinal Study

**DOI:** 10.1155/2024/9688717

**Published:** 2024-08-21

**Authors:** Diva Lugassy, Gil Ben-Izhack, Sara Zissu, Rotem Shitrit Lahav, Ophir Rosner, Nir Uziel, Sarit Naishlos, Asaf Shely

**Affiliations:** ^1^ Department of Orthodontics The Maurice and Gabriela Goldschleger School of Dental Medicine Sackler Faculty of Medicine Tel Aviv University, Tel Aviv 6997801, Israel; ^2^ Department of Oral Rehabilitation The Maurice and Gabriela Goldschleger School of Dental Medicine Sackler Faculty of Medicine Tel Aviv University, Tel Aviv 6997801, Israel; ^3^ Department of Pediatric Dentistry The Maurice and Gabriela Goldschleger School of Dental Medicine Tel-Aviv University, Ramat-Aviv 69978, Israel

## Abstract

**Background:**

The aim of this study was to evaluate the changing levels of stress among dental students during 8 months of a basic manual skills course in the preclinical year and to examine the association between stress and dental performance.

**Methods:**

A longitudinal study was conducted in the 2023 academic year in a total of 58 (male = 17 and female = 41; mean age = 26.43, range 22–33) undergraduate dental students at Tel Aviv University of dentistry during their fourth year of study. Depression Anxiety Stress Scale (DASS-21) and Dental Environment Stress (DES) questionnaires were used to assess the psychological well-being and the severity of DASS symptoms experienced by the students. The students' dental performances were assessed using two manual tests on plastic teeth. The questionnaires and the manual tests were used at three periods of time, T0, T1, and T2. Wilcoxon signed-rank tests were performed to compare the DASS scores and DES stressors of dental students between T0, T1, and T2. Kendall's nonparametric correlations were calculated to investigate the relationships of DES stressors and depression, anxiety, and stress scores with manual performance.

**Conclusions:**

The perception of high stress by dental students is due to the stressful education process of the preclinical year. There is an inverse correlation between the lower level of anxiety and the increase level of dental performance with 74% of the variance in dental performance explained by the anxiety score. Work-related stressors such as manual skills might reduce dental performance in contrast to non-work-related factors such as financial obligations, personal issues, and family factors, which might increase student dental performance.

## 1. Introduction

Dentistry is a medical profession that requires various capabilities such as fine motor skills [1], hand–eye coordination [2], and spatial perception [3] in order to perform precise dental procedures. During the preclinical year of the dentistry degree, students train on a conventional simulator (a phantom head), which simulates dental procedures on plastic teeth by using dental instruments and turbines [4]. For dental students, achieving a level of competence and safety in fine motor skills is a challenging task. Due to the complexity of manual skill acquisition, dental students find the dental education process highly stressful [5], which can lead to psychological disturbance in the form of depression, anxiety, and a loss of self-confidence [6, 7]. Chronic stress due to continuing negative persistent stressors may affect the well-being of students in the form of fatigue, tiredness, sleep disturbance, abdominal disturbances [8], and temporomandibular disorders (TMD) [9].

High stress levels and their association with other illnesses in dental students have been the subject of many studies [9, 10, 11, 12]. Owczarek et al. [9] showed that there is a positive relationship between the high manifestation of TMD and the higher level of psychoemotional states among dental students. Naidu et al. [10] showed that dental students are placed under many stressful situations that may lead to high levels of psychological disturbance. Al-Sandook et al. [11] concluded that stress among dental students produces a significant elevation in blood pressure and that the risk factors for other diseases increase if the stressor persists.

While there is a consensus in the literature regarding the stress stimulation of health problems among dental students, the correlation between stress and dental students' clinical performance and cognitive ability is controversial [12]. Albandar et al. [5] concluded that stress did not affect students' dental performance; Sanders and Lushington [13] found little support for the association between increased stress and reduced dental performance; and Lin et al. [14] showed that stress levels negatively correlated with dental performance, finding that, as the stress level increased, the grade point average (GPA) decreased. According to a systematic review by Plessas et al. [12] regarding the impact of stress on dentists' clinical performance, there is a gap in the literature, as there are no prospective experimental studies that compare stressful situations with nonstressful situations in terms of the association between stress and performance.

Like dental students, surgeons are subjected to many stressors which might compromise their competence on surgical performance that directly influence the patient safety [15]. In the study of Arora et al. [16], surgeons suggested that stress during surgery procedure impairs their judgement and decision-making and breaks down the teamwork communication which potentially affect the patient safety. Wetzel et al. [17] confirm that surgery is a highly pressured field with many stressors that affect performance adversely. Those studies investigated the impact of acute stressors which have episodic event with discreet beginning and ending on the clinical performance mostly by interview with the surgeons with lack of data concerning the relationship of chronic ongoing stressful conditions and level of performance [18].

The gap in the literature regarding the important association of prolonged harmful stress and the level of clinical performance raise the awareness for developing this longitudinal study. We hypothesized that there is an association between prolonged stress during the process of manual acquisition and the reduced dental performance of dental students during their preclinical year of study. Therefore, this study aimed to examine the changing levels of stress among dental students during 8 months of a basic manual skills course in the preclinical year and to evaluate the association between stress and dental performance.

## 2. Materials and Methods

A longitudinal study was conducted in the 2023 academic year in a total of 58 undergraduate dental students at Tel Aviv University of dentistry during their fourth year of study. The students constituted the entire student class that year. The dental students were in their preclinical year of practicing on a phantom head, and none of them had received any previous manual dexterity training.

The students' participation was completely voluntary after the explanation of the goals of the research and its importance by the principal investigator. The experiment was conducted according to the guidelines and approved by the Institutional Ethics Committee of Tel Aviv University (document no. 0005853-1, date approved 4 January 2023), and all participants signed an informed consent document before commencing the study. For anonymity, each participant was randomly assigned an identifying number known only to the principal investigator.

### 2.1. Psychological Well-Being Evaluation

Psychological well-being was assessed using the Depression Anxiety Stress Scale (DASS-21), a self-rated questionnaire that assesses the severity of DASS symptoms experienced in the previous week. DASS- 21 item is a modified and shorter version of the original version of DASS-42. It is a reliable and accepted globally for screening instrument of severity measurement of depression, anxiety, and stress symptoms [19].

Items are scored on a 4-point scale: did not apply to me at all (0), applied to me to some degree or some of the time (1), applied to me to a considerable degree or a good part of the time (2), and applied to me very much or most of the time (3). The dental students used the 4-point scale to rate their symptoms during the previous week. The DASS scores were calculated by summing the scores of seven statements relevant to each subscale: stress, anxiety, and depression. The scores for each subscale were then categorized to indicate the level of severity of each condition ranging from normal, mild, moderate, severe, and extremely severe according to the cut points suggested by Loviband [20]. Severe and extremely severe symptom scores were recorded as clinically significant for increased risk for severe illness [21].

### 2.2. Dental Environment Stressor Evaluation

To determine the perceived sources of stress, the Dental Environment Stress (DES) questionnaire was used, which is relevant to dental school education [22]. This questionnaire identifies and quantifies stressors specific to dental students. It contains 25 items and five response categories (1 = not stressful to 5 = very stressful). The items belong to seven subdomains: faculty and administration (Questions 9, 12, and 18), academics (Questions 1–4), manual skills (Questions 6 and 10), financial obligations (Question 21), patient care (Questions 5, 7, 8, and 11), personal problems (Questions 13–17, 22, and 25), and family (Questions 19, 20, and 22–24).

### 2.3. Dental Performance

Before performing the dental tasks by the students, they underwent a lecture regarding the concepts of cavity preparation and demonstration of the cavity by clinical instructors. The students' dental performances were assessed via the grades given on manual tests performed during a prosthodontic dentistry course at the phantom laboratory: (1) plastic tooth preparation for a fixed partial denture (FPD) and (2) a temporary acrylic crown. Tooth preparation was performed on plastic tooth no. 46 mounted on the phantom head simulator, and a temporary acrylic crown was performed upon it.

Due to poor examiner reliability by human subjectivity compounded by a paucity of objective parameters, three instructors provided the grades using several common criteria, such as the inclination of tooth preparation, the long axis of the preparation, morphology, and the adaptation of the temporary crown to the tooth preparation. The examiners were guided how to fill a checklist with specific criteria to result in standardized data for evaluation dental performance.

For each criterion, the instructor chose between four possibilities: excellent, good, fair but not failure, and failure. The grades are on a continuous scale of 0–100, where the passing grade is 60. As the student identification numbers were coded, the instructors were unaware of the individual students' results. The study was blinded in the sense that the principal investigator who conducted the tests was blind to the students' manual test results in the prosthodontic dentistry course.

### 2.4. Course of Study (Figure 1)

The experiment was performed at three time points before the theoretical final exams period of each semester that might influence the level of stress of the students:

- T0: 4 months after the begining of the phantom course
- T1: 4 months after T0 and 2 weeks before the final exam
- T2: 1 month after T1

All dental students (*N* = 58) participated at T0 and T1. At T2, 12 students were randomly selected to evaluate the association between stress and dental performance under nonstressful conditions (1 month after T1 and 2 weeks after the final exam) after finishing the phantom course as a control group; thus, we avoided selection bias.

During T0 and T1, all students (*N* = 58) performed the same order of testing:

- Completed the Depression Anxiety Stress Scale (DASS-21) questionnaire (5 min)
- Completed the Dental Environment Stress (DES) questionnaire (5 min)
- Performed two dental performance tests on a plastic tooth at the phantom laboratory: tooth preparation and a temporary acrylic crown (80 min)

During T2, the students (*N* = 12) performed the same order of testing:

- Completed the Depression Anxiety Stress Scale (DASS-21) questionnaire (5 min)
- Completed the Dental Environment Stress (DES) questionnaire (5 min)
- Performed one dental performance test on a plastic tooth at the phantom laboratory: tooth preparation (40 min)

During the phantom course, clinical instructors serve as models for observation and demonstration of the perfect performance of the dental tasks before physical practice. During training, one of the important roles of the instruction team of the phantom course is to provide objective terminal external feedback after completion of dental task.

### 2.5. Statistics

All analyses were performed using SPSS, version 20 (IBM Corp., Armonk, NY). Significant statistical differences were defined as *p* < 0.05.

Wilcoxon signed-rank tests were performed to compare the DASS scores and DESS stressors of dental students between T0, T1, and T2.

Kendall's nonparametric correlations were calculated to investigate the relationships of DES stressors and depression, anxiety, and stress scores with dental performance.

Three linear regression models, using the enter method, were conducted to examine the strength and combination of the explanatory stress variables on the clinical performance.

A sensitivity power analysis using G^*∗*^Power was performed to calculate which effect size our study is sensitive enough to detect.

## 3. Results

A total of 58 (male = 17 and female = 41; mean age = 26.43, range 22–33) undergraduate dental students at Tel Aviv University of dentistry during their fourth year of study participated in this study. A Wilcoxon signed-rank test (matched pairs) with 58 participants would be sensitive to detect effect sizes of Cohen's *f* = 0.38 with 80% power (*α* = 0.05). This corresponds to medium effect size.

Kolmogorov–Smirnov tests indicated a nonnormal distribution of the dependent variables (students' scores in DASS, DES, and manual performance), *p* < 0.05 (Table 1).

### 3.1. Dental Environment Stressor Evaluation

At the end of practicing in the phantom lab (T1), the stressor scores of the students significantly increased compared to T0 for stress due to amount of classwork (*p*=0.006), stress due to difficulty of classwork (*p*=0.0005), stress due to examinations and grades (*p*=0.033), stress due to patient care responsibilities (*p*=0.02), stress due to patients' attitudes toward me (0.005), stress due to patient's attitudes toward dentistry (*p*=0.022), and stress due to atmosphere created by clinical professors (*p*=0.0005), faculty and administration (*p*=0.003), academics (*p*=0.009), and patient care (*p*=0.047) and in the total DESS score (*p*=0.027) (Table 1).

In order to evaluate the associations of the stressor scores under nonstressful conditions, 12 students were randomly selected 1 month after T1 (2 weeks after the final exam at the phantom course, T2). Before analyzing their stressors, Mann–Whitney tests and chi-square tests were performed to compare data of those 12 students with the rest of the students (*n* = 46) in terms of age, gender, and stressor scores for preventing confounding that would have affected the internal validity of the study. No statistically significant differences were found between the 12 students and the rest of the students with regard to age (*p*=0.301), gender (*p*=0.307), and stressor at T0 and T1, respectively, due to faculty and administration (*p*=0.479, *p*=0.900), patient care (*p*=0.684, *p*=0.867), academics (*p*=0.307, *p*=0.179), manual skill (*p*=0.607, *p*=0.420), financial obligation (*p*=0.156, *p*=0.099), personal problem (*p*=0.165, *p*=0.135), and family (*p*=0.278, *p*=0.824).

We identified those 12 students and followed their stressor scores throughout the course of the study. At T1, the stressor scores of the students significantly increased compared to T0 for stress due to patients' attitudes toward me (*p*=0.05) and stress due to patient's attitudes toward dentistry (*p*=0.031). At T2, the stressor scores significantly decreased compared to T1 for stress due to the amount of classwork (*p*=0.048), faculty (*p*=0.05), and academics (*p*=0.034) and in the total DESS score (*p*=0.034) (Table 1).

### 3.2. Psychological Well-Being Evaluation

The overall prevalence of depression, anxiety, and stress was 48.3%, 55.2%, and 52.6% at T0; 69%, 60.3%, and 65.5% at T1; and 41.7%, 25%, and 33.3% at T2, respectively. At T1, significantly more students experienced DASS symptoms of depression (*p*=0.005) and anxiety (*p*=0.06) (Table 2). At the end of practicing in the phantom lab (T1), the mean DASS scores of depression (*p*=0.003), anxiety (*p*=0.004), and stress (*p*=0.026) levels increased significantly compared to those at T0 (Table 3).

In order to evaluate the associations of DASS scores under nonstressful conditions, 12 students were randomly selected 1 month after T1 (T2). No significant differences were found between the 12 students and the rest of the students (*n* = 46) with regard to DASS scores of depression (*p*=0.588, *p*=0.551), anxiety (*p*=0.985, *p*=0.923), and stress (*p*=0.331, *p*=0.736) at T0 and T1 respectively.

We identified those 12 students and followed their DASS scores throughout the course of the study. At T1, significantly more students experienced DASS symptoms of depression (*p*=0.02), while at T2, the DASS symptoms reduced for depression (*p*=0.05), anxiety (*p*=0.01), and stress (*p*=0.018). At T1, the mean depression (*p*=0.022) increased significantly, while 1 month after T1 (T2), the depression (*p*=0.036), anxiety (*p*=0.007), and stress (*p*=0.041) decreased significantly compared to T1 (Table 3).

### 3.3. Association of Dental Performance and Stress

The DASS scores of depression, anxiety, and stress of the dental students at T0, T1, and T2 were not significantly correlated with clinical performance, except for the anxiety score at T2, which was significantly and inversely correlated with tooth preparation (*r* = −0.718, *p*=0.003) (Table 4). Furthermore and although a small sample size at T2 might compromise the results, a linear regression analysis using the enter variable selection method using anxiety score at T2 variable that revealed significant correlations in the previous correlation test was applied. The anxiety score at T2 explained 74% of the variance in the tooth preparation grade at T2 (*R*^2^ = 0.746, *ß* = −0.864, *p*=0.0005) and produced a significant model (*p* = 0.0005).

No significant differences were found between the 12 students and the rest of the students (*n* = 46) regarding tooth preparation (*p*=0.098, *p*=0.144) and acrylic crown (*p*=0.726, *p*=0.250) at T0 and T1, respectively.

There was no significant correlation between all seven subdomain stressors and the total DES score with dental performance at T0, T1, and T2, except for the positive correlation between the financial obligations stressor and tooth preparation (*r* = 0.253, *p*=0.011) at T0, the financial obligations stressor and tooth preparation at T1 (*r* = 0.253, *p*=0.011), the patient care stressor and temporary crown at T1 (*r* = 0.302, *p*=0.002), personal problems and temporary crown at T1 (*r* = 0.229, *p*=0.002), the family stressor and temporary crown at T1 (*r* = 0.201, *p*=0.039), and the total DES score and temporary crown at T1 (*r* = 0.224, *p*=0.019) and the inverse correlation between the manual skill stressor and tooth preparation at T2 (*r* = −0.513, *p*=0.032) (Table 5). Furthermore, a linear regression analysis using the enter variable selection method using independent variables (patient care at T1, personal problem at T1, and family stressor at T1) that revealed significant correlations in the previous correlation test was applied. Those three variables explained 32% of the variance in the crown preparation grade at T1 (*R*^2^ = 0.321) and produced a significant model (*p*=0.0005) with the higher influence of the personal stressor (*ß* = 0.350) compared to patient care (*ß* = 0.270) and family stressor (*ß* = 0.01). Another linear regression analysis was made by using the manual stressor at T2 and revealed a significant model (*p*=0.04) with this variable explained 35% of the variance in tooth preparation at T2 (*R*^2^ = 0.358, *ß* = −0.598, *p*=0.04).

## 4. Discussion

While most of the studies that examined the link between stress and the well-being or clinical performance of dental students were cross-sectional, we conducted a prospective, longitudinal study in order to evaluate the changing levels of stress among dental students during 8 months of the acquisition of manual skills in the preclinical year and to examine the association of chronic stress with their dental performance. Studies from the healthcare literature have demonstrated that stress may affect memory [23], communication skills [24], diagnostic skills [25], and psychomotor performance [26]. In our study, we showed inverse correlation between the lower level of anxiety and the increase level of dental performance with 74% of the variance in dental performance explained by the anxiety score at T2.

The results of the present study indicate that dental students at Tel Aviv University experienced a high level of stress during their preclinical year, with the peak of stress at the end of the year (T1), 2 weeks before the final exam of the phantom course, which determines whether the students will progress to the subsequent year of study and treat patients. Interestingly, 1 month after T1 and 2 weeks after the final exam, the stress level decreased significantly which allowed us to postulate that the perception of high stress by dental students is due to the stressful education process of dentistry studies. Alzahem A. M. et al. [27] showed in their systematic review that one of the main strategies for the stress management of dental students is decreasing the number of stressors and workload pressure due to examinations and requirements. In our study, during the preclinical year, the number of stressors relating to faculty and administration, academics, and patient care increased significantly, and after finishing the phantom course, most of those stressors decreased. Unsurprisingly, considering the high amount of knowledge that the students are required to master in addition to the difficulty of acquiring the manual skills required to perform dental procedures, the students often consider themselves as incompetent for the task. We therefore suggest that dental education schools need to reduce the workload and academic requirements in order to decrease undesirable high levels of stress of dental students, as these levels have the potential to become chronic and might affect their professional lives as dentists. Our results are consistent with those of other studies that found that the areas identified as most stressful by dental students were student–faculty relationships, academics, and clinical workload stressors [26, 28, 29, 30].

Interestingly, according to our results and confirm by regression analysis, the stressors related to financial obligations, patient care, and personal and family problems had a positive impact on dental performance and are therefore considered motivation factors; this is in contrast to the manual skills stressor, which had a negative relationship with dental performance. In contrast, our previous study did not show correlation between lateral pinch modulation test and dental students performance [31]. According to the literature [32], individuals with a strong goal orientation can be more vulnerable to work-related stressors. A high focus on work-related goals has been associated with a low interest in other goals, such as family and relationships, which modified the negative impact of work-related stressors. The cumulative outcome might cause a psychological and mental reaction. The goal-setting theory [33] claims that motivation factors can predict job performance the more challenging the goal is. Academic performance is dependent on multiple factors, two of which are stress and motivation. According to this study, work-related stressors such as manual skills reduced dental performance, whereas non-work-related factors such as financial obligations and personal and family factors increased the motivation of the dental students to achieve better dental performance.

The study had some main limitations. At Tel Aviv University, the number of students is small, and the instructors' evaluation is subjective due to paucity of objective parameters. Therefore, smaller amount of students participated in the present study compared to other studies, and three instructors provided the grades for dental performance. In addition, following the research pattern of the present study, only a part of the students were randomly selected at T2 to perform one task only, which affect the accuracy of this longitudinal study. The accuracy of estimators in regression analysis is affected by the small sample size which might lead to inaccurate results.

Future longitudinal research on the link of stress and clinical performance with the entire research group follow throughout the three time point periods is required to increase the awareness toward the harmful potential of reduced clinical performance by dentists due to their mental disturbance that subsequently might affect patient safety.

## 5. Conclusions

The perception of stress by dental students increased due to the stressful education process of the preclinical year. There is an inverse correlation between the lower level of anxiety and the increase level of dental performance with 74% of the variance in dental performance explained by the anxiety score. Work-related stressors such as manual skills might reduce dental performance in contrast to non-work-related factors such as financial obligations, personal issues, and family factors, which might increase student dental performance.

## Figures and Tables

**Figure 1:**
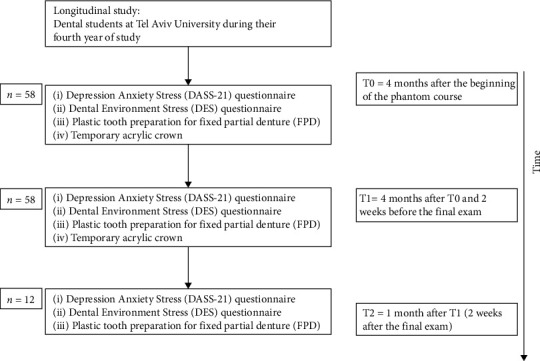
Course of the study.

**Table 1:** Mean scores and SD of Dental Environment Stress (DES) questionnaire items rated on a 5-point scale at three periods of time: T0 (*N* = 58), T1 (*N* = 58), and T2 (*N* = 12).

	Stress factors in dental educational environment	T0 *n* = 58	T1 *n* = 58	T2 *n* = 12
		Mean ± SD	Mean ± SD	Mean ± SD
1	Stress due to amount of classwork	3.25 ± 1.08	3.74 ± 1.29	2.33 ± 1.23
2	Stress due to difficulty of classwork	2.94 ± 1.20	3.13 ± 1.36	2.58 ± 0.90
3	Stress due to examinations and grades	3.32 ± 1.31	3.62 ± 1.33	2.83 ± 1.26
4	Stress due to peer competition	2.37 ± 1.16	2.48 ± 1.34	1.92 ± 0.90
5	Stress due to patient care responsibilities	2.15 ± 1.22	2.65 ± 1.55	1.92 ± 1.16
6	Stress due to difficulty in learning clinical procedures	2.70 ± 1.19	2.60 ± 1.26	2.58 ± 0.79
7	Stress due to patients' attitudes toward me	1.49 ± 0.98	1.96 ± 1.30	1.67 ± 0.98
8	Stress due to patient's attitudes toward dentistry	1.57 ± 1.06	2.02 ± 1.31	1.67 ± 0.88
9	Stress due to atmosphere created by clinical professors	2.48 ± 1.27	3.29 ± 1.36	2.25 ± 1.21
10	Stress due to difficulty in learning precision manual skills required in preclinical and laboratory practice	3.10 ± 1.20	2.86 ± 1.31	2.17 ± 0.71
11	Stress due to reliability professional dental laboratories in prompt return of cases	1.98 ± 1.16	1.98 ± 1.01	1.67 ± 0.88
12	Stress due to administrative response to student needs	2.61 ± 1.30	2.87 ± 1.31	2.58 ± 0.90
13	Stress due to roommate relationships	1.60 ± 0.89	1.44 ± 0.99	1.25 ± 0.45
14	Stress due to dating relationships	1.72 ± 1.01	1.72 ± 1.21	1.92 ± 0.99
15	Stress due to alcohol usage	1.06 ± 0.36	1.06 ± 0.55	1.08 ± 0.28
16	Stress due to drug usage	1.06 ± 0.41	1.00 ± 0.49	1.08 ± 0.28
17	Stress due to reconsideration of dentistry as proper carrier	1.93 ± 1.10	2.00 ± 1.35	2.25 ± 1.05
18	Stress due to fear of flunking out of school	2.63 ± 1.40	2.60 ± 1.50	2.25 ± 1.28
19	Stress due to marriage relationship	1.50 ± 1.06	1.46 ± 1.06	1.75 ± 1.42
20	Stress due to childcare	1.16 ± 0.49	1.17 ± 0.79	1.00 ± 0.00
21	Stress due to financial responsibilities	3.39 ± 1.34	3.27 ± 1.51	3.58 ± 0.90
22	Stress due to personal physical health	2.65 ± 1.33	2.96 ± 1.49	2.75 ± 1.35
23	Stress due to physical health of other family members	2.48 ± 1.40	2.79 ± 1.56	2.83 ± 1.52
24	Stress due to parent–student relationship	1.96 ± 1.24	2.06 ± 1.28	2.00 ± 0.85
25	Stress due to other personal problems	2.75 ± 1.30	2.91 ± 1.38	3.08 ± 1.08
	Total score	57.13 ± 14.9	59.96 ± 21.06	53.00 ± 11.19
	Faculty and administration	2.58 ± 1.03	2.92 ± 1.13	2.36 ± 0.75
	Academics	2.97 ± 0.96	3.24 ± 1.14	2.41 ± 0.71
	Manual skills	2.91 ± 1.10	2.73 ± 1.17	2.37 ± 0.71
	Financial obligations	3.39 ± 1.34	3.27 ± 1.51	3.58 ± 0.90
	Patient care	1.78 ± 0.91	2.15 ± 1.17	1.72 ± 0.73
	Personal problems	1.83 ± 0.52	1.87 ± 0.75	1.91 ± 0.54
	Family	1.93 ± 0.74	2.09 ± 0.86	2.06 ± 0.75

**Table 2:** Outcome of DASS-21 among dental students at T0, T1, and T2.

Category	T0 *n* (%)	T1 *n* (%)	T2 *n* (%)
Depression			
Normal	30 (51.7)	18 (31.0)	7 (58.3)
Mild	13 (22.4)	13 (22.4)	1 (8.3)
Moderate	3 (5.2)	12 (20.7)	3 (25)
Severe	7 (12.1)	9 (15.5)	1 (8.3)
Extremely severe	5 (8.6)	6 (10.3)	0 (0)
Anxiety			
Normal	26 (44.8)	23 (39.7)	9 (75)
Mild	13 (22.4)	7 (12.1)	1 (8.3)
Moderate	6 (10.3)	5 (8.6)	1 (8.3)
Severe	4 (6.9)	6 (10.3)	0 (0)
Extremely severe	9 (15.5)	17 (29.3)	1 (8.3)
Stress			
Normal	28 (47.4)	20 (34.5)	8 (66.7)
Mild	7 (12.3)	7 (12.1)	3 (25)
Moderate	11 (19.3)	14 (24.1)	1 (8.3)
Severe	7 (12.3)	9 (15.5)	0 (0)
Extremely severe	5 (8.8)	8 (13.8)	0 (0)

**Table 3:** Mean and SD of Depression Anxiety and Stress Scale (DASS) symptoms, tooth preparation, and temporary acrylic crown of dental students at T0, T1, and T2.

	Mean ± SD depressive level	Mean ± SD anxiety level	Mean ± SD stress level	Mean ± SD tooth preparation	Mean ± SD temporary acrylic crown
T0	5.60 ± 5.04	4.89 ± 4.36	8.66 ± 4.88	71.33 ± 7.89	60.27 ± 17.65
T1	6.94 ± 4.59	6.36 ± 4.97	10.00 ± 5.14	67.63 ± 18.23	61.89 ± 21.18
T2	4.33 ± 3.22	2.66 ± 3.17	5.75 ± 3.04	84.42 ± 4.88	—

**Table 4:** Association of depression, anxiety, and stress scores with dental performance among dental students at T0 (*n* = 58), T1 (*n* = 58), and T2 (*n* = 12).

	Dental performance	Depression	Anxiety	Stress
T0	Tooth preparation	*r* = −0.035	*r* = 0.095	*r* = 0.010
	Temporary acrylic crown	*r* = −0.060	*r* = 0.002	*r* = −0.101

T1	Tooth preparation	*r* = 0.004	*r* = −0.009	*r* = 0.058
	Temporary acrylic crown	*r* = 0.075	*r* = −0.011	*r* = 0.020

T2	Tooth preparation	*r* = −0.38	*r* = −0.718 ^*∗∗*^	*r* = −0.374
		—	*p*=0.003	—

^*∗∗*^Kendall's tau-b correlation is significant at the 0.01 level.

**Table 5:** Association of DES stressors and dental performance among dental students at T0 (*n* = 58), T1 (*n* = 58), and T2 (*n* = 12).

	Dental performance	Faculty and administration	Academics	Manual skills	Financial obligations	Patient care	Personal problems	Family	Total DESS Score
T0	Tooth preparation	*r* = 0.027	*r* = −0.022	*r* = −0.087	*r* = 0.228 ^*∗*^ *p*=0.032	*r* = 0.007	*r* = 0.173	*r* = −0.136	*r* = 0.090
	Temporary acrylic crown	*r* = −0.020	*r* = −0.035	*r* = −0.037	*r* = 0.209	*r* = 0.116	*r* = 0.169	*r* = 0.176	*r* = 0.067

T1	Tooth preparation	*r* = 0.045	*r* = −0.021	*r* = −0.007	*r* = 0.253 ^*∗*^ *p*=0.011	*r* = 0.148	*r* = 0.030	*r* = 0.029	*r* = 0.070
	Temporary acrylic crown	*r* = 0.065	*r* = 0.011	*r* = 0.071	*r* = 0.166	*r* = 0.302 ^*∗∗*^ *p*=0.002	*r* = 0.229 ^*∗*^ *p*=0.018	*r* = 0.201 ^*∗*^ *p*=0.039	0.224 ^*∗*^ *p*=0.019

T2	Tooth preparation	*r* = −0.25	*r* = −0.164	*r* = −0.513 ^*∗*^	*r* = −0.074	*r* = −0.410	*r* = −0.048	*r* = −0.066	*r* = −0.371
		—	—	*p*=0.032	—	—	—	—	—

^*∗∗*^Kendall's tau-b correlation is significant at the 0.01 level.  ^*∗*^Correlation is significant at the 0.05 level.

## Data Availability

The data presented in this study are available on request from the corresponding author.
